# High-Accuracy ncRNA Function Prediction via Deep Learning Using Global and Local Sequence Information

**DOI:** 10.3390/biomedicines11061631

**Published:** 2023-06-03

**Authors:** Alessandro Orro, Gabriele A. Trombetti

**Affiliations:** Institute for Biomedical Technologies, National Research Council (ITB-CNR), 20054 Segrate, Italy; gabriele.trombetti@itb.cnr.it

**Keywords:** artificial intelligence, bioinformatics, genomics, ncRNA, function prediction, machine learning

## Abstract

The prediction of the biological function of non-coding ribonucleic acid (ncRNA) is an important step towards understanding the regulatory mechanisms underlying many diseases. Since non-coding RNAs are present in great abundance in human cells and are functionally diverse, developing functional prediction tools is necessary. With recent advances in non-coding RNA biology and the availability of complete genome sequences for a large number of species, we now have a window of opportunity for studying non-coding RNA biology. However, the computational methods used to predict the non-coding RNA functions are mostly either scarcely accurate, when based on sequence information alone, or prohibitively expensive in terms of computational burden when a secondary structure prediction is needed. We propose a novel computational method to predict the biological function of non-coding RNA genes that is based on a collection of deep network architectures utilizing solely ncRNA sequence information and which does not rely on or require expensive secondary ncRNA structure information. The approach presented in this work exhibits comparable or superior accuracy to methods that employ both sequence and structural features, at a much lower computational cost.

## 1. Introduction

In recent years, growing access to massive transcriptome sequencing technologies has led to the discovery of an increasing number of novel transcripts from various species. The majority of these transcripts result in non-coding ribonucleic acid (ncRNA) molecules, short sequences of RNA that, with the exception of a small number of junk RNAs, are involved in a variety of important biological processes such as gene regulation [1], alternative splicing [2], and deoxyribonucleic acid (DNA) replication [3]. Additionally, ncRNAs are implicated in various diseases [4], either as biomarkers (e.g., abnormal expression levels) or in the pathophysiology of cancer, cardiovascular, and neurological disorders [5].

Due to complementary sequence regions (G–C and A–U pairs), ncRNA molecules fold into complex secondary structures that strongly characterize their function, although the biological mechanisms are not fully understood. The class of long ncRNAs (longer than 200 nucleotides) includes intergenic, intronic, sense, and antisense ncRNAs, depending on their genomic location and transcription direction. These ncRNAs have a role in transcription, translation, and splicing [6,7]. Short ncRNAs (shorter than 200 nucleotides) are responsible for several vital biological functions, such as protein assembly by the ribosomal RNA (rRNA), amino acid transfer into the rRNA site by transfer RNA (tRNA), messenger RNA (mRNA) splicing by small nuclear RNA (snRNA), and gene regulation by microRNAs (miRNAs).

As much as 75% of the human genome is transcribed into RNA [8], but protein-coding regions account for approximately just 1.22–1.5% [9,10], leaving a vast majority of transcripts with unknown function; despite this, the functional landscape of ncRNAs remains largely uncharted. Understanding the functions of these ncRNAs is therefore crucial for deciphering the regulatory networks governing cellular behavior and dysfunction in diseases. Enhancing the accessibility and accuracy of ncRNA function prediction has the potential to accelerate research in ncRNA biology, drive the development of ncRNA-based diagnostics and therapeutics, and ultimately contribute to the advancement of precision medicine [11,12].

In a genome-wide context, annotated databases can be leveraged using computational methods for rapid and accurate ncRNA classification and for linking functions to potential diseases. One of the best known of such databases is Rfam [13], which contains a massive manually and literature-based curated collection of non-coding RNA sequences grouped into 3444 families with common ancestors. For each family, it provides raw data sequences, multiple alignments (seed alignments), secondary structures, and statistical computational models that can be used to search for potential homologs of an unclassified sequence.

Other relevant ncRNA databanks exist: lncRNAdb [14], which reports several annotations (such as function, expression, and subcellular localization) for a large set of long ncRNAs; miRBase [15] and deepBase [16], which are based on deep sequencing data; and ncRNAdb [17], which also provides ncRNA information for archaea.

According to a well-established bioinformatics paradigm, biomolecules (such as proteins) having similar sequences or structures are likely to share a similar biological function. Applying this paradigm to ncRNAs resulted in various computational tools for automatic functional classification.

While methods based solely on sequence information are simple and execute rapidly, they can fail to detect distant homologs and can result in a high false negative rate. BLAST [18] and BLAT [19] are widely known examples. Such tools operate via rapidly comparing a query sequence against a massive dataset of annotated reference sequences, and with an algorithm that can allow for small mismatches in exchange for a penalty, result in an e-value score. The query sequence is predicted to have the same function as the first hit if the e-value score for the first hit is lower than an acceptable threshold.

Other approaches use machine learning techniques to build predictive models. In this category, the use of Convolutional Neural Network (CNN) [20] or recurrent Long Short-Term Memory Network (LSTM) [21] architectures allows for good prediction accuracy.

On the other hand, methods that incorporate (or rely entirely on) structural features can achieve the best accuracy, but their application may be limited by the prohibitively high computational costs associated with secondary structure prediction. In this category, GraPPLE [22] uses features obtained from a graph representation of secondary structure only to map a ncRNA to one of the 41 classes taken from the Rfam family using a Support Vector Machine (SVM). nRC [23] extracts subgraph structural features from IPknot’s [24] predicted secondary structure and uses them to train a Convolutional Neural Network (CNN).

The idea of combining both primary and secondary information is the strength of tools such as INFERNAL [25], which predicts unknown classes using statistical covariance models (CMs) built from primary and secondary structure; EDeN [26], which exploits graph kernels; and RNAGCN [27], which uses Graph Convolutional Networks and a graph representation of ncRNA.

In recent years, Deep Learning (DL) has emerged as a leading machine learning methodology due to its ability to generate multiple encodings of input features at many (“deep”) levels of representation. In particular, the ability of CNNs to process sequences is well suited to the treatment of biological sequences and has already been adopted [21] both for functional classification and ncRNA recognition within the genome [28].

In an attempt to minimize overfitting, which is a known problem for CNNs, these are frequently structured as a cascade of small window modules (rank between three and five base pairs); nonetheless, this approach may limit the neural network’s ability to capture long-range or global information embedded into the sequence. On the other hand, while LSTM recurrent networks are capable of processing entire sequences, they suffer from a number of disadvantages, including high computational costs [29] and difficulty in training [30], which are ultimately due to their complex architecture, substantially limiting their application to relatively short sequences.

Although advanced machine learning architectures should be capable of inferring knowledge from data on their own, the direct inclusion of additional domain knowledge into the training process is very appealing [31,32], not only for increased accuracy and computational speed, but also for obtaining simpler and more interpretable models. This strategy is also useful when extremely simple interpretations are used. In the context of Artificial Neural Networks, domain knowledge can be inserted using three main approaches: (1) in the encoding of the input features, (2) in the choice of the loss function (mainly as regularization terms), and (3) in the network architecture.

In this research, we tackle the problem of ncRNA function prediction using a set of relatively simple and computationally fast Deep Neural Network architectures that leverage a combination of global and local information derived solely from ncRNA sequences. Our approach exhibits comparable or superior accuracy to methods that incorporate both sequence and structural information or that use more advanced Deep Neural Networks, and the advantage is also maintained in the case of simple and small network architectures. Moreover, our approach offers relevant advantages in terms of training speed. The main idea is that while standard CNN layers process local information, a set of parallel neural modules enhances the prediction through injecting global information into the final output level.

## 2. Materials and Methods

### 2.1. Dataset

In order to facilitate comparisons with other methods, we used a common publicly available dataset [23] composed of 8920 ncRNA sequences (6320 training and 2600 test) classified into 13 functional classes: miRNA, 5S rRNA, 5.8S rRNA, ribozymes, CD-box, HACA-box, scaRNA, tRNA, Intron gpI, Intron gpII, IRES, leader, and riboswitch. The average length of the sequences is 158, and the difference in length within classes cannot be used as a feature of the ML method due to a large standard deviation. The full description of the dataset is reported in Table 1, including the length distribution (mean ± standard deviation).

### 2.2. Architecture

**Figure 1:**
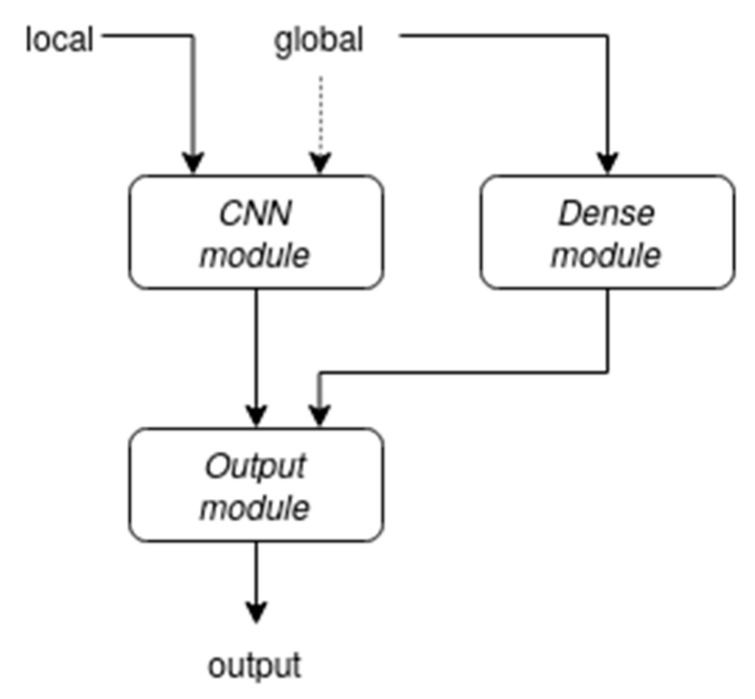
An overview of the proposed architecture. The connection represented by the dashed arrow was not present in the final version of our network.

The template architecture, as depicted in Figure 1, is composed of three main modules, each fed with a different type of information.

**Figure 2:**
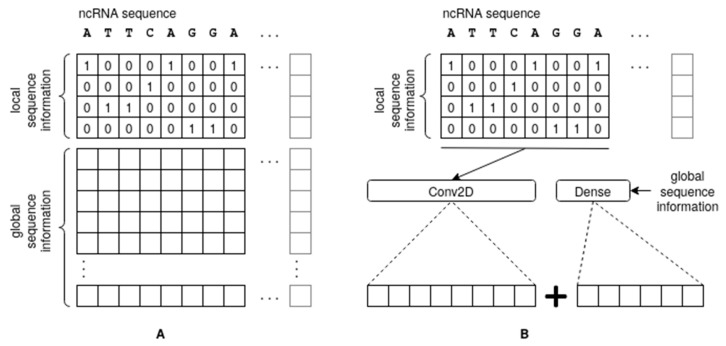
Introduction of local sequence information (**A**) and global information (**B**) into the architecture. The global information can also be introduced in (**A**), as shown, but this was not done in the final version of our network.

The first module is used to process the local sequence information and maps the main bases A, C, T, and G using 4-bit one-hot encoding (see Figure 2A); other symbols in the dataset have been encoded as a zero vector for simplicity, as they were very rare. The resulting 2D matrix is processed via a 2D CNN, followed by a series of 1-dimensional CNNs. The use of simple one-hot encoding (a k-mer with *k* = 1) does not add complexity to the pattern, leaving the responsibility of finding the best combination of features to the CNN. Numerous more sophisticated encoding schemes have been proposed in the literature in an attempt to capture the structure of ncRNA, such as space-filling curves [33], which compress the sequence into a 2D or 3D array following specific schemes. Such arbitrary encoding schemes attempt to artificially introduce spatial proximity into the feature space in a way that has no relevant biological meaning. Our approach is supported by experimental evidence demonstrating superior performance of k-mer encoding with *k* = 1 [21].

The second module processes a vector of global sequence information evaluated for each sequence and feeds it into a Multiple Layer Perceptron (MLP) of dense layers having rectifier linear unit (ReLU) activation functions and a dropout layer for overfitting reduction. While any type of global feature can be employed at this level, in our work, we tested the following three types: class propensities, as computed using a set of k-mer based statistics (with varying values of small *k*) evaluated across the entire sequence; a simple and weak Bayesian classifier trained on medium-length sequence patterns; and, finally, class affinity scores derived from an instance-based similarity approach. Moreover, the global information generated via these modules can be injected into the Deep Neural Network architecture either at the input module, stacked onto the one-hot encoding of the input (Figure 2A), or concatenated with the output (Figure 2B).

The third (output) module is primarily responsible for integrating (concatenating) the outputs coming from the other two modules and using this as an input to produce a final output classification. The final layer is a softmax dense layer with a neuron for each class.

### 2.3. Global Information Representation

One of the main aspects of our proposed method is the incorporation of global statistics for ncRNA molecules into the predictive machine learning models. This information, which would hardly be captured during the training through observing only a short subsequence, provides a global context that significantly increases classification performance and training curve speed.

We used three sets of global information:

1. K-mer statistics using small values of *k*;
2. Class propensities derived from medium-length pattern occurrences; and
3. Instance-based class propensities.

These three types of information can be injected into the Deep Neural Network architecture independently, either into the input encoding or at the output level, as previously mentioned.

#### 2.3.1. Small K-mers

Given a small number *k* (typical values from 2 to 6), a k-mer is a string of length *k* that could be contained, as a substring, within a biological sequence. The presence (with multiplicity) of all possible $4^{k}$ k-mers in a sequence s can be encoded as an integer array of counters of length $4^{k}$, subsequently normalized as an array of $4^{k}$ floats of sum 1. Such an array can then be injected into the Neural Network using the approach described above.

**Figure 3:**
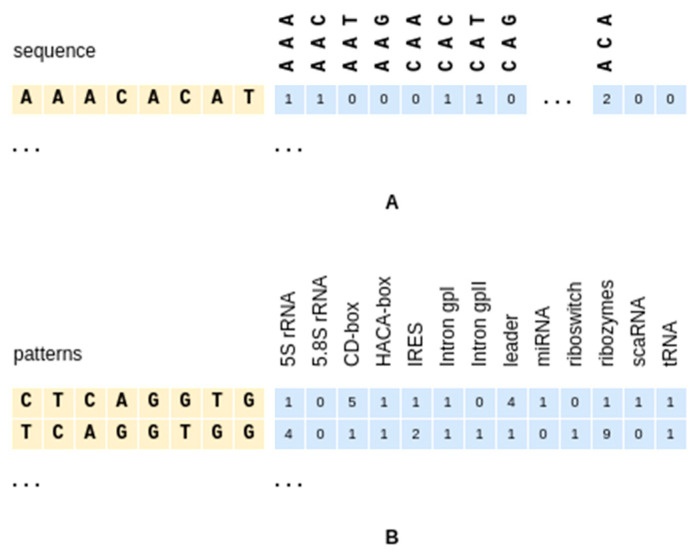
Encoding of the k-mers (**A**) and sequence patterns (**B**). Both are shown before normalization.

The probability of an input sequence s of length *len*(*s*) containing, one or multiple times, a k-mer *kmer*(*i*) as a substring, can be described, in first approximation (statistical independence of the bases of the sequence, perfect randomness) as follows:

(1) $$P\left(kmer\left(i\right)|s\right)=1-{\left(1-\frac{1}{4^{k}}\right)}^{len\left(s\right)-k+1}\;i\in \left\{1,2,\ldots ,4^{k}\right\}$$

where *len*(*s*) − *k* + 1 is the count of the (contiguous and overlapping) k-mers in an input sequence of length *len*(*s*). An example for *k* = 3 is shown in Figure 3A.

#### 2.3.2. Sequence Patterns

Given a sequence pattern *p* of length *L* (typical values 8 to 11), the class propensity for a sequence *s*, which contains the pattern *p* as a substring, to belong to class *c* is evaluated as the normalized number of sequence matches per class, as found in the training dataset, for that pattern.

(2) $$P\left(class\left(s\right)=c\;|\;p\;\mathrm{in}\;s\right)=\frac{N\left(c,p\right)}{N\left(p\right)}$$

(3) $$\underset{c\in \mathbb{C}}{\sum }P\left(class\left(s\right)=c\;|\;p\;\mathrm{in}\;s\right)=1$$

where *N*(*c*, *p*) is the count of sequences in class *c* containing the pattern *p*, *N*(*p*) is the total count of sequences containing the pattern *p*, and *ℂ* is the set of classes of the classification problem.

A table of all *P*(*class*(*s*) = *c*|*p* in *s*) of a training dataset can be easily computed, and can then be used to obtain a vector of class propensities for a test sequence (Figure 3B) as

(4) $$P\left(class\left(s\right)=c\right)=\frac{\sum_{p\in \mathbb{P}}\mathrm{count}\_\mathrm{occurr}\left(p,s\right)\cdot P\left(class\left(s\right)=c\;|\;p\;\mathrm{in}\;s\right)}{\sum_{p\in \mathbb{P}}\mathrm{count}\_\mathrm{occurr}\left(p,s\right)}\;c\in \mathbb{C}$$

where *ℙ* is the set of available patterns and *count*_*occurr*(*p*,*s*) is a function counting the occurrences of string *p* contained within string *s*. The resulting *P*(*class*(*s*) = *c*)|*c*∈ℂ is the vector encoding of the global information to be injected into the Neural Network.

Both types of global encoding (small k-mers and sequence patterns) extract information from sub-sequence patterns. The difference is that while medium-length subsequences (around 10 bases) have a higher predictive power, they generate millions of entries, rendering the prediction problem intractable, when used as independent features. On the other hand, short subsequences (up to 6 bases) result in fewer features (4096 for *k* = 6) that could be used to feed a predictive model but merely give contextual information. Hence, we addressed them differently, employing two distinct Neural Network modules.

#### 2.3.3. Instance-Based Class Propensities

A BLAST search of a target ncRNA sequence against an annotated ncRNA dataset allows the straightforward transfer of the class of the best hits to the target sequence. This approach is used to generate class propensities and feed them as global data into the machine learning model during training. The encoding resembles the “Sequence Patterns” case as previously described. The pairs of sequences are compared using both BLAST and global alignment searches (*gap*_*penalty* = −1, substitution matrix = identity).

### 2.4. Neural Network Configuration

**Figure 4:**
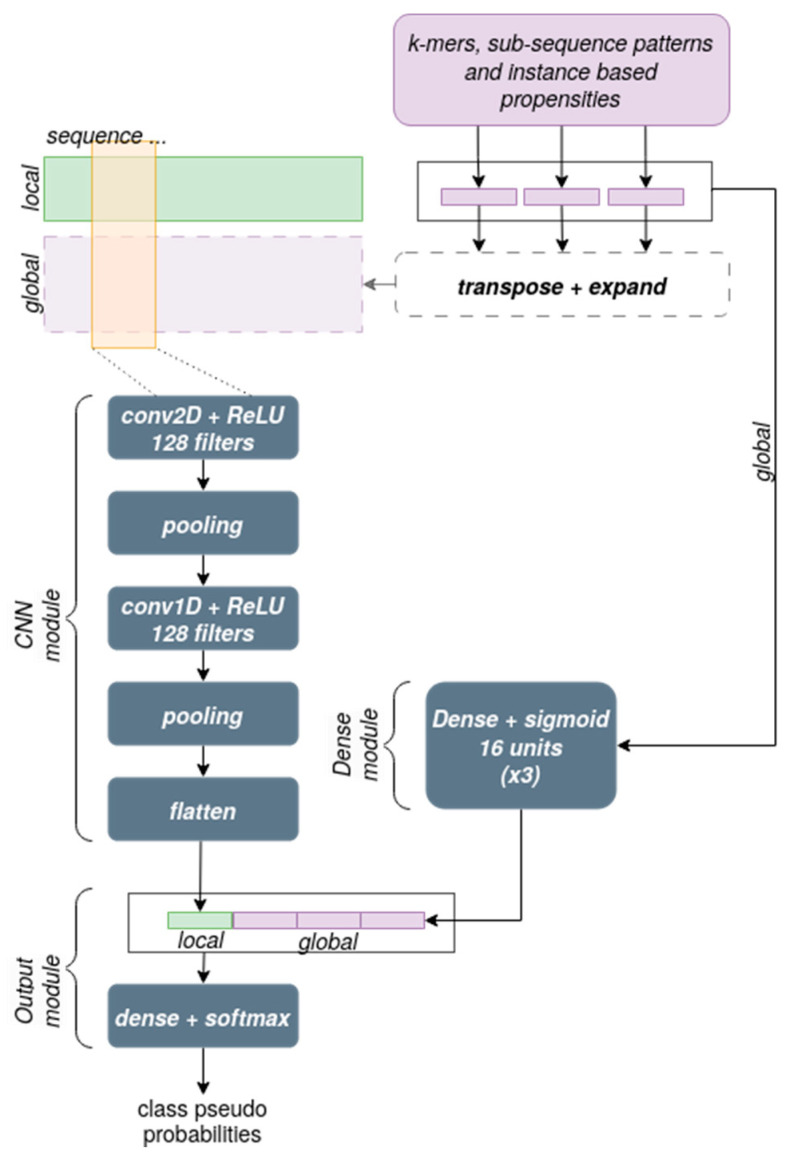
Details of the network architecture. The elements outlined by dashed lines were not included in the final version of our network.

The network architecture used in the experiments is shown in Figure 4. The module devoted to processing the sequence information consists of a 2-dimensional Convolutional Neural Network in which the first dimension corresponds to the sequence left to right and the second dimension corresponds to the 4 channels for the bases in one-hot encoding (local information), with an added channel for each type of global information that is used at this level. Cascading from that, a 1-dimensional Convolutional Network module is applied. Both convolutional levels have 128 filters, a kernel size of 5 along the sequence, a stride of 2, a ReLU activation function, and are both followed by an average pooling layer that calculates a downsampled feature map through calculating the mean value of the feature map patches.

The module devoted to integrating the global information at the output level performs a simple concatenation operation that occurs just before the final output layer, which consists of a standard Dense layer with a sigmoid activation function and a number of neurons equal to the number of classes, as is commonly done in multi-class problems.

The models were trained with an Adam optimization backpropagation algorithm (learning rate 0.5 × 10^−3^) set to update the weights, splitting the training dataset into a batch of size 32 at each epoch.

The Categorical Cross Entropy is used as a loss function:

(5) $$CCE=-\underset{i\in classes}{\sum }t_{i}\cdot log\left(p_{i}\right)$$

where *t* is the one-hot encoding of the true label and $p_{i}$ is the corresponding pseudoprobability of the predicted class.

The accuracy and *F*1 scores are monitored during the training on both the training and test sets, and reported in the comparison report (described later):

(6) $$ACC=\frac{TP+TN}{TP+TN+FP+FN}$$

(7) $$F1=\frac{2\cdot P\cdot R}{P+R}$$

where *TP*, *TN*, *FP,* and *FN* are true positive, true negative, false positive, and false negative, respectively, and *P* and *R* are the precision and recall, respectively defined as

(8) $$precision=P=\frac{TP}{TP+FP}$$

(9) $$recall=R=\frac{TP}{TP+FN}$$

## 3. Results

The performance metrics in this section were derived through aggregating the outcomes of eight separate experiments. For each of those experiments, the neural network was trained (backpropagation) using a random 80% subset of the 6320-sequence “training” set from Fiannaca et al.’s nRC [23], while the remaining random 20% of the “training” set from nRC was used as a validation set to determine the most accurate training epoch. The additional set of 2600 sequences from nRC was used as the final test set to obtain the performance metrics reported here.

A first series of experiments was conducted to assess the impact, in terms of accuracy and training speedup, of incorporating global information into the architecture of a Neural Network model. A second series of experiments was performed to compare the proposed solution with other state-of-the-art prediction systems that represent a range of machine learning strategies, such as Graph Convolutional Neural Networks (GCNNs) and Long Short-Term Memory (LSTM) Recurrent Neural Networks (RNNs), as well as different uses of input information and encoding methods, including primary and secondary protein structure information and subgraph structural features.

The best configuration resulted in being the one with the global information concatenated just before the output layer and without it being injected at the first layer. It appears that the two modules can learn the features more accurately if the two types of information are kept separated as long as possible, only merging them towards the end. In fact, a module that processes local information will train slower compared to one that uses global information from some kind of pre-trained input.

The global information parametrization employed was as follows: *L* = 11 (length of the pattern), *k* = 6 (length of the k-mer words), *gap*_*penalty* = −1, and an identity substitution matrix (score 1 for match and 0 for mismatch) for the instance-based encoding. Such L and K values appeared to perform best, as higher lengths matched too infrequently and produced very sparse data matrices (overfitting), while lower values tended to be too generic and less discriminative. The chosen gap_penalty and identity scoring matrix are typical parameter values for the alignment of RNA or DNA sequences.

### 3.1. Impact of Introducing Global Information

In order to evaluate the effectiveness of introducing various types of global information to a simple Convolutional Neural Network (CNN) classifier using only one-hot encoding, a set of experiments was conducted. Multiple strategies for integrating global information were tested, as described in the Materials and Methods section. The final and best performing architecture included all three types of global information concatenated together in the last layer of the Neural Network.

The outcomes are shown in Table 2. A simple Convolutional Neural Network (CNN) that did not incorporate global information yielded an initial accuracy of 86.03 percent. Through gradually providing CNN with more global data, we were able to increase its accuracy to 97.20%. Initially, the incorporation of the instance-based information alone yielded the greatest improvement, achieving 95.45% accuracy. The next most advantageous addition was the incorporation of k-mers, which increased the accuracy to 96.87%. Finally, the incorporation of sequence patterns allowed us to attain the final 97.20% accuracy rate.

A parameter called “step75” was defined, which represents the point during the backpropagation training at which the accuracy reaches 75% on the training set. This parameter serves as a measure of the speedup that can be achieved through introducing global information into the training process. As shown in Table 2, the introduction of global information progressively increases the speedup (as reflected in lower values of step75). Overall, these experiments demonstrate the significant improvement that can be obtained through incorporating global information into the architecture of a Neural Network model.

### 3.2. Comparison with Other Methods

In order to speed up the comparison, we performed the same experiment as [21] (named “CNN improved” here and in Table 5 of [21]), which uses a powerful LSTM-based neural network approach to predict ncRNA function using exactly the same dataset. The reported results are the best results as taken from the cited literature and include the accuracy and F1 score as metrics. In particular, our comparison includes the following methods: EDeN, nRC, RNAGCN, and CNN improved.

As our proposed method is able to predict ncRNA function with 97.20% accuracy and a 97.11 F1 score, it appears to outperform all the other cited methods from the literature using the same dataset (Table 3), albeit, in one case, by a small margin.

**Figure 5:**
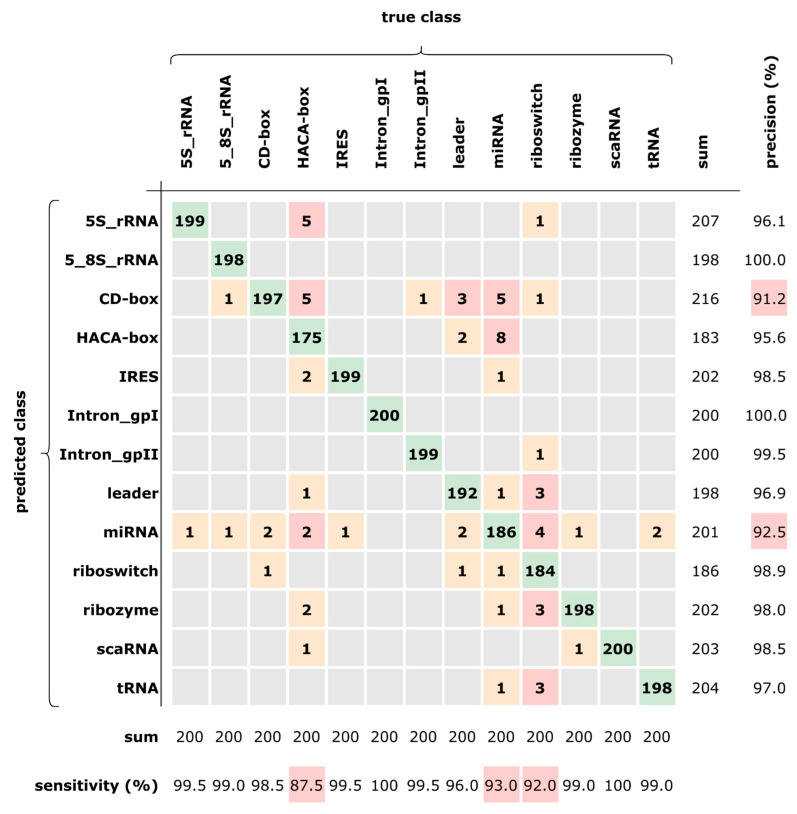
Confusion matrix of the prediction for our best network.

The confusion matrix in Figure 5 shows the predictions over the test dataset (2600 sequences) and has been computed so as to evaluate the per-class accuracy. The best accuracy (≥99%) has been obtained in seven classes, whereas the three classes CD-box, miRNA, and HACA-box resulted in being more difficult to predict (lower sensitivity). This could be explained by the fact that HACA-box and CD-box belong to the same superclass “small nucleolar RNAs” (snoRNAs) and hence share many similarities. Moreover, miRNA resulted in being particularly hard to predict, being often confused with other classes having the same percentages (but not with Intron_gpI, Intron_gpII, and scaRNA).

Computational performance: Both the training and inference phases executed very fast due to our simple and non-recurrent network architecture. The training executed at 22 ms/step, or about 7 s per epoch (316 steps/epoch), while the inference timing was 260 ns/sequence (K20 GPU on a dual Xeon E5-2667 server).

All experiments, except for the above benchmark, were run on a Linux Ubuntu 22.04 server, with an Intel i7 2.8 GHz CPU, 32 GB RAM, and an NVIDIA Quadro P2000 GPU.

## 4. Discussion

Non-coding RNAs (ncRNAs) are RNA molecules that do not encode proteins but have been shown to have a variety of functions in cells, such as gene expression regulation, transcriptional and post-transcriptional regulation, and structural support roles. Numerous tools for predicting the functional class of specific ncRNAs using primary sequence and structural information (particularly secondary structure) and advanced machine learning techniques, such as Recurrent Neural Networks and Graph Convolutional Networks, have been developed in the scientific literature. This research suggests that the accuracy of ncRNA functional class prediction can be improved through incorporating “global domain specific information”, which refers to information regarding the wider biological context in which the ncRNA molecule is found.

In this context, secondary structure information could be effectively leveraged due to its strong correlation with biological function; however, determining it with sufficient accuracy is both computationally expensive and challenging. Deep Learning architectures are renowned for their ability to automatically extract complex features from unprocessed data and develop encodings that enable accurate predictions but are difficult and time-consuming to train. LSTM networks, in particular, are able to capture complex temporal patterns, making them ideal for datasets consisting of sequentially ordered features.

Hence, our proposed method aims to improve upon previous strategies for predicting the functional class of non-coding RNA (ncRNA) molecules through employing a set of relatively small Deep Neural Network architectures that can incorporate both global and local information derived from the primary sequences of the ncRNA molecules. Preprocessed sequence features that may not be easily captured using ordinary convolutional or LSTM models have been included, so as to assist the network in identifying complex sequence features.

The present approach leverages existing well-established techniques for representing and generalizing sequence information in bioinformatics, such as sequence patterns and k-mers (short DNA or RNA sequences of a fixed length), and the typical functional transfer paradigm in bioinformatics, which suggests that biomolecules with similar primary sequences or functional patterns are likely to have the same biological function. Through injecting these preprocessed sequence features into the Neural Network architecture, the proposed method seeks to improve and outperform existing techniques for predicting ncRNA functional classes.

The proposed architecture can be viewed from a computer science and machine learning standpoint as a combination of two advanced machine learning techniques: instance-based learning and cascade classification. 

In instance-based learning, rather than developing a general rule or model, the system learns through storing and analyzing specific examples (instances from the training set). This method does not require the construction of a general model of the data beforehand, as it predicts the class of new instances via comparing them to the stored examples, and because of this, it can be very computationally efficient. Instance-based learning is also capable of dealing with high-dimensional data, which can result in a remarkable degree of precision when the number of features or variables is large. However, the method can be less reliable when the number of stored instances is small or when the instances are not representative of the overall data distribution. A cascade classifier is a machine learning technique that involves dividing the classification process into a series of stages, in which each stage determines the instance’s class based on the output of the previous stage. The decision-making process can then be incrementally improved through adding additional cascade stages.

In this view, the proposed approach would be a two-stage cascade architecture, with the first stage employing a standard Convolutional Neural Network and the second stage employing a set of three weak classifiers: one trained on long sequence patterns, one trained on short k-mers, and a third one using an instance-based approach.

## 5. Conclusions

Non-coding RNA (ncRNA), RNA molecules that do not code for proteins, directly perform a variety of critical cellular processes such as regulating gene expression, mediating RNA splicing, controlling cell differentiation, controlling cell metabolism and development, and maintaining genomic integrity. Determining ncRNA functions is therefore pivotal to gaining a comprehensive understanding of cellular and organismal biology as well as identifying potential therapeutic targets for human diseases.

Computer-based prediction of ncRNA functions is an open problem with partial solutions which our work aimed to enhance.

In our approach, we used the power of Deep Learning to predict the functional properties of publicly available ncRNA sequences. Our approach did not use an NN to classify ncRNA sequences into structures, but instead operated on sequences directly, for increased computational performance and increased reliability of the training phase. Our architecture compensated for a lack of ncRNA structural information through using contextual information, and in order to do this, a set of specialized weak classifiers, simple DNNs, were utilized to inspect and interpret different aspects of global context information.

Leveraging the above-mentioned structure, our architecture achieved what appears to be the highest accuracy among numerous competing Deep Learning methods from the literature for ncRNA classification.

Additionally, our work showed that through proper construction of the NN layers, very high accuracy can be achieved with relatively low network complexity, also resulting in high computational performance and an architecture that could also be run on commodity hardware.

## Figures and Tables

**Table 1:** Non-coding RNA functional classes of the dataset used for the experiments.

ncRNA Class	Description	Length
miRNA	microRNA, small single-stranded non-coding ribonucleic acid (ncRNA) mainly involved in RNA silencing and gene expression post-transcriptional regulation.	10 ± 41
5S rRNA	5S ribosomal RNA plays a role in the stabilization of the ribosome, of which it is one of the components	119 ± 9
5.8S rRNA	5.8S ribosomal RNA, similar to the 5S rRNA, is a component of the ribosome in eukaryotes; it plays a role in protein translation	153 ±15
ribozymes	Ribozymes (ribonucleic acid enzymes) have enzymatic behavior in catalyzing specific biochemical reactions	260 ± 158
CD-box	C/D box guide snoRNA, a subclass of small nucleolar RNAs (snoRNAs) involved in the methylation of RNA molecules	106 ± 42
HACA-box	H/ACA box snoRNA, a subclass of small nucleolar RNAs (snoRNAs) involved in the pseudouridylation of RNA molecules	140 ± 35
scaRNA	Small Cajal body-specific RNA plays a guiding role in the methylation and pseudouridylation of RNA polymerase II transcribed spliceosomal RNA	174 ± 76
tRNA	Transfer RNA plays a role in translation, binds the ribosome, and transfers a specific amino acid of a growing polypeptide chain	78 ± 13
Intron gpI	Group I Intron is a type of ribozyme able to extract itself from another RNA molecule; it has catalytic activity and is involved in intron splicing	342 ± 99
Intron gpII	Group II intron is similar to Group I but uses a different type of splicing reaction	96 ± 26
IRES	Internal Ribosome Entry Site is an RNA involved in protein synthesis	235 ± 103
leader	Leader RNA is a term that refers to the region of a messenger RNA that precedes the starting codon and has an important role in the regulation of translation of a transcript	125 ± 30
riboswitch	Riboswitch is a region of an mRNA molecule that regulates the corresponding protein encoding through the action of a specific binding ligand	142 ± 50

**Table 2:** Comparison of different configurations *.

Type of Global Information	Step75	F1 Score (%)	Accuracy (%)
Local-only	1433	85.96	86.03
KM	18	91.55	91.64
SP	681	89.97	90.10
IB	461	95.46	95.49
KM + SP	14	94.11	94.22
KM + IB	14	96.87	96.90
SP + IB	27	95.75	95.80
KM + SP + IB	9	97.11	97.20

* Comparison of a basic CNN classifier (local-only) using solely local sequence information against more powerful Deep Learning Neural Networks whose architecture adds increasing levels of global information: k-mers (KM), Sequence Patterns (SP) and Instance-Based (IB), as described in the Materials and Methods section.

**Table 3:** Comparison of the proposed architecture against four state-of-the-art approaches.

Method	F1 Score (%)	Accuracy (%)
EDeN (*)	65	67
nRC	81.81	81.66
RNAGCN	85.73	85.61
CNN improved (*)	96	96
This work	97.11	97.20

* The EDeN Accuracy and F1 score when used on the Fiannaca et al.’s nRC [23] dataset are taken from [21]. Article [21] does not declare decimals for F1 score and Accuracy for EDeN and CNN improved.

## Data Availability

Not applicable.

## References

[1] Wery M., Kwapisz M., Morillon A. (2011). Noncoding RNAs in gene regulation. Wiley Interdiscip. Rev. Syst. Biol. Med..

[2] Romero-Barrios N., Legascue M.F., Benhamed M., Ariel F., Crespi M. (2018). Splicing regulation by long noncoding RNAs. Nucleic Acids Res..

[3] Ge X.Q., Lin H. (2014). Noncoding RNAs in the regulation of DNA replication. Trends Biochem. Sci..

[4] Esteller M. (2011). Non-coding RNAs in human disease. Nat. Rev. Genet..

[5] Lekka E., Hall J. (2018). Noncoding RNAs in disease. FEBS Lett..

[6] Ma L., Bajic V.B., Zhang Z. (2013). On the classification of long non-coding RNAs. RNA Biol..

[7] Ransohoff J.D., Wei Y., Khavari P.A. (2018). The functions and unique features of long intergenic non-coding RNA. Nat. Rev. Mol. Cell Biol..

[8] Djebali S., Davis C.A., Merkel A., Dobin A., Lassmann T., Mortazavi A., Tanzer A., Lagarde J., Lin W., Schlesinger F. (2012). Landscape of transcription in human cells. Nature.

[9] The ENCODE Project Consortium (2012). An Integrated Encyclopedia of DNA Elements in the Human Genome. Nature.

[10] International Human Genome Sequencing Consortium (2001). Initial sequencing and analysis of the human genome. Nature.

[11] Tian H., Zhou C., Yang J., Li J., Gong Z. (2017). Long and short noncoding RNAs in lung cancer precision medicine: Opportunities and challenges. Tumor Biol..

[12] Smith E.S., Whitty E., Yoo B., Moore A., Sempere L.F., Medarova Z. (2022). Clinical Applications of Short Non-Coding RNA-Based Therapies in the Era of Precision Medicine. Cancers.

[13] Griffiths-Jones S., Bateman A., Marshall M., Khanna A., Eddy S.R. (2003). Rfam: An RNA family database. Nucleic Acids Res..

[14] Amaral P.P., Clark M.B., Gascoigne D.K., Dinger M.E., Mattick J.S. (2011). lncRNAdb: A reference database for long noncoding RNAs. Nucleic Acids Res..

[15] Kozomara A., Griffiths-Jones S. (2014). miRBase: Annotating high confidence microRNAs using deep sequencing data. Nucleic Acids Res..

[16] Yang J.-H., Shao P., Zhou H., Chen Y.-Q., Qu L.-H. (2010). deepBase: A database for deeply annotating and mining deep sequencing data. Nucleic Acids Res..

[17] Szymanski M., Erdmann V.A., Barciszewski J. (2007). Noncoding RNAs database (ncRNAdb). Nucleic Acids Res..

[18] Altschul S.F., Gish W., Miller W., Myers E.W., Lipman D.J. (1990). Basic local alignment search tool. J. Mol. Biol..

[19] Kent W.J. (2002). BLAT—The BLAST-Like Alignment Tool. Genome Res..

[20] Chantsalnyam T., Lim D.Y., Tayara H., Chong K.T. (2020). ncRDeep: Non-coding RNA classification with convolutional neural network. Comput. Biol. Chem..

[21] Noviello T.M.R., Ceccarelli F., Ceccarelli M., Cerulo L. (2020). Deep learning predicts short non-coding RNA functions from only raw sequence data. PLoS Comput. Biol..

[22] Childs L., Nikoloski Z., May P., Walther D. (2009). Identification and classification of ncRNA molecules using graph properties. Nucleic Acids Res..

[23] Fiannaca A., La Rosa M., La Paglia L., Rizzo R., Urso A. (2017). nRC: Non-coding RNA Classifier based on structural features. BioData Min..

[24] Sato K., Kato Y., Hamada M., Akutsu T., Asai K. (2011). IPknot: Fast and accurate prediction of RNA secondary structures with pseudoknots using integer programming. Bioinformatics.

[25] Nawrocki E.P., Eddy S.R. (2013). Infernal 1.1: 100-fold faster RNA homology searches. Bioinformatics.

[26] Navarin N., Costa F. (2017). An efficient graph kernel method for non-coding RNA functional prediction. Bioinformatics.

[27] Rossi E., Monti F., Bronstein M., Liò P. (2019). NcRNA Classification with Graph Convolutional Networks. arXiv.

[28] Liu X.-Q., Li B.-X., Zeng G.-R., Liu Q.-Y., Ai D.-M. (2019). Prediction of Long Non-Coding RNAs Based on Deep Learning. Genes.

[29] Khalil K., Eldash O., Kumar A., Bayoumi M. (2019). Economic LSTM Approach for Recurrent Neural Networks. IEEE Trans. Circuits Syst. II: Express Briefs.

[30] Pascanu R., Mikolov T., Bengio Y. (2013). On the Difficulty of Training Recurrent Neural Networks. arXiv.

[31] Dash T., Chitlangia S., Ahuja A., Srinivasan A. (2022). A review of some techniques for inclusion of domain-knowledge into deep neural networks. Sci. Rep..

[32] Muralidhar N., Islam M.R., Marwah M., Karpatne A., Ramakrishnan N. Incorporating Prior Domain Knowledge into Deep Neural Networks. Proceedings of the IEEE International Conference on Big Data.

[33] Cannon J.W., Thurston W.P. (2007). Group invariant Peano curves. Geom. Topol..

